# ARPC1B binds WASP to control actin polymerization and curtail tonic signaling in B cells

**DOI:** 10.1172/jci.insight.149376

**Published:** 2021-12-08

**Authors:** Gabriella Leung, Yuhuan Zhou, Philip Ostrowski, Sivakami Mylvaganam, Parastoo Boroumand, Daniel J. Mulder, Conghui Guo, Aleixo M. Muise, Spencer A. Freeman

**Affiliations:** 1 Program in Cell Biology and; 2 SickKids Inflammatory Bowel Disease Centre, Peter Gilgan Centre for Research and Learning, The Hospital for Sick Children, Toronto, Ontario, Canada.; 3 Department of Biochemistry and; 4 Department of Pediatrics, University of Toronto, Toronto, Ontario, Canada.

**Keywords:** Cell Biology, Immunology, Calcium signaling, Cytoskeleton, Genetic diseases

## Abstract

Immune cells exhibit low-level, constitutive signaling at rest (tonic signaling). Such tonic signals are required for fundamental processes, including the survival of B lymphocytes, but when they are elevated by genetic or environmental causes, they can lead to autoimmunity. Events that control ongoing signal transduction are, therefore, tightly regulated by submembrane cytoskeletal polymers like F-actin. The actin-binding proteins that underpin the process, however, are poorly described. By investigating patients with ARPC1B deficiency, we report that ARPC1B-containing ARP2/3 complexes are stimulated by Wiskott Aldrich Syndrome protein (WASP) to nucleate the branched actin networks that control tonic signaling from the B cell receptor (BCR). Despite an upregulation of ARPC1A, ARPC1B-deficient cells were not capable of WASP-mediated nucleation by ARP2/3, and this caused the loss of WASP-dependent structures, including podosomes in macrophages and lamellipodia in B cells. In the B cell compartment, ARPC1B deficiency also led to weakening of the cortical F-actin cytoskeleton that normally curtails the diffusion of BCRs and ultimately resulted in increased tonic lipid signaling, oscillatory calcium release from the endoplasmic reticulum (ER), and phosphorylated Akt. These events contributed to skewing the threshold for B cell activation in response to microbial-associated molecular patterns (MAMPs). Thus, ARPC1B is critical for ARP2/3 complexes to control steady-state signaling of immune cells.

## Introduction

ARPC1B deficiency is a recently described, rare genetic disorder that causes a Wiskott-Aldrich Syndrome–like (WAS-like) disease in children (1–3). It follows an autosomal recessive inheritance pattern and is clinically marked by leukocytoclastic vasculitis, thrombocytopenia, immune defects, eczema, otitis media, and bloody eosinophilic colitis. ARPC1B deficiency in T cells impairs immune synapse formation and the targeted release of lytic granules, ARP2/3-dependent processes, which helps to explain the reported immune defects (3, 4). Similar to WAS, ARPC1B-deficient patients also have increases in circulating CD19^+^ cells, soluble IgE, sIgA, and autoantibodies, which is suggestive of an intrinsic, autoactivated B cell compartment (1, 5, 6). A role for ARPC1B in immune cells beyond T lymphocytes, however, has not been described.

ARPC1B is a member of the ARP2/3 complex, which is composed of 7 subunits: ARP2, ARP3, ARPC1, ARPC2, ARPC3, ARPC4, and ARPC5 (7). As an assembled complex, ARP2/3 nucleates the polymerization of daughter filaments that branch at regular angles from preexisting mother filaments (7). Stimulation of the complex by nucleation promotion factors (NPFs) (e.g., WAS protein [WASP]) serves as a rate-limiting step in this process (8, 9). Isoforms of the ARP2/3 complex subunits, as well as the NPFs that stimulate ARP2/3, show recent evolutionary divergence and tissue-specific expression patterns. *ARPC1* is one example of an ARP2/3 complex subunit with 2 isoforms in humans (10); the genomes of most other eukaryotes contain just a single gene for each ARP2/3-complex subunit, including *ARPC1*. The two *ARPC1* isoforms in humans share 67% sequence identity, and while ARPC1A has a largely ubiquitous expression pattern, ARPC1B is uniquely and highly expressed in immune cells (11). In the organisms that express one *ARPC1* isoform, the subunit can be essential for life (12); however, an understanding for the emergence of its isoforms in humans remains unknown.

Spatially regulated branching of F-actin networks is crucial for immune cell functions, including cell migration, spreading, and adhesion, and for specialized types of endocytosis, including phagocytosis (13–16). Though less appreciated, actin polymers also regulate constitutive or “tonic” signaling in immune cells by compartmentalizing and tethering receptors, coreceptors, and inhibitory transmembrane phosphatases in the plane of the plasma membrane (17–19). In B lymphocytes, the actin cytoskeleton, along with tetraspanins, maintain the B cell receptor (BCR) largely separated from its coreceptor CD19 (18). Disrupting the membrane-associated actin cytoskeleton and proteins that tether it to the plasmalemma, as occurs when receptors are engaged by arrays of cognate antigens (20, 21), leads to the coalescence of the receptor into microclusters together with CD19 to stimulate PI3K signaling (18). In the steady state, the submembrane actin cytoskeleton is thought to control the threshold for B cell activation by such mechanisms while also permitting the tonic, survival signals that emanate from the BCR (22). Such control is paramount to B cell responses, as is exemplified by the fact that mutations in actin-regulating proteins lead to autoimmunity (5, 23).

Herein, we find that WASP operates concertedly with ARPC1B-containing ARP2/3 complexes to nucleate the cortical branched actin networks that regulate BCR diffusion and tonic signaling. We report that mutations in ARPC1B lead to dysfunction of the B cell compartment, leading to high numbers of circulating transitional B cells in patients. Taken together, our findings establish a mechanism where the loss of an ARP2/3 complex subunit leads to immunological pathology via its regulation of steady-state signaling and helps to explain the etiology of patients with ARPC1B deficiency.

## Results

### ARPC1B deficiency causes the loss of steady-state cortical F-actin and Arp2/3-mediated F-actin polymerization in B cells.

**Figure 1:**
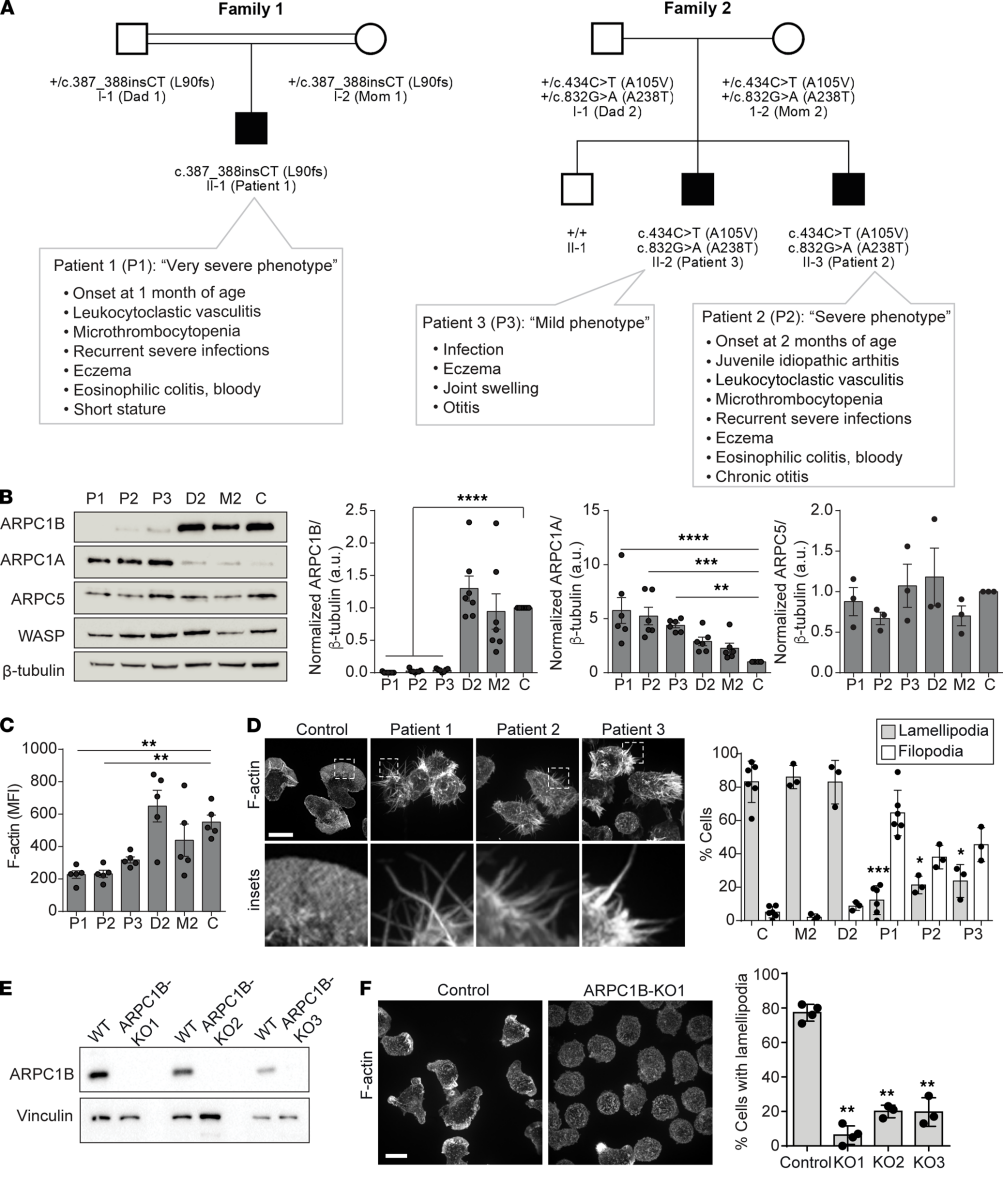
ARPC1B deficiency causes dysregulation of the cortical actin cytoskeleton in B cells, despite increased ARPC1A expression. (**A**) Pedigrees of families affected by ARPC1B deficiency. Clinical presentations have been updated since the initial report (1). Sample names are indicated in parentheses. (**B**) Immunoblot of ARPC1B patient LCL whole cell lysates and densitometry quantification; *n* = 4. The correlation between ARPC1B and ARPC1A density was fitted using a centered second order polynomial (quadratic) regression analysis. (**C**) Total cellular F-actin in unstimulated cells as stained by Alexa Fluor 488–conjugated phalloidin and measured by flow cytometry; *n* = 5. For **B** and **C**, data were analyzed using a 1-way ANOVA with Dunnett’s post hoc test compared with control. See also Supplemental Figure 1. (**D**) LCLs stained with Alexa Fluor 488–phalloidin (F-actin) in suspension (left). Cells were quantified for lamellipodia or filopodia from 5 fields, containing > 5 cells (right); *n* = 3. Dots represent the means from each *n*. Scale bar: 10 μm. (**E**) Immunoblot of Ramos B cell ARPC1B-KO clones. (**F**) Ramos WT and ARPC1B-KO cells stained with Alexa Fluor 488–phalloidin (F-actin) in suspension (left). Cells with lamellipodia were quantified as in **D** (right); *n* = 3 or 4. Scale bar: 10 μm. *P < 0.05; **P < 0.01; ***P < 0.001; ****P < 0.0001.

Three patients with ARPC1B deficiency were originally described in Kahr et al., and the clinical presentations of these patients have been updated since the initial report (Figure 1A) (1). In addition to analyzing their primary circulating B cells (see later), we also generated immortalized lymphoblastoid cell lines (LCL) using B cells from these patients, their parents, and additional control donors in order to obtain sufficient material to perform biochemical analyses. By immunoblotting lysates made from the LCLs, we confirmed the loss of ARPC1B in the patient-derived B cells and found that this was concomitant with an upregulation in the expression of the ARPC1A (Figure 1B). Despite the upregulation of ARPC1A, the total F-actin density of the cells determined by measuring the mean fluorescence intensity of Alexa Fluor 488–phalloidin was decreased 2- to 3-fold in those derived from patients versus healthy cells (Figure 1C and Supplemental Figure 1; supplemental material available online with this article; https://doi.org/10.1172/jci.insight.149376DS1). Branched F-actin networks, like those nucleated by ARP2/3, drive membrane ruffling and lamellipodia formation. At rest, we found that patient-derived cells were unable to form lamellipodia and, instead, formed filopodia-like protrusions (Figure 1D). To confirm these effects are attributed to ARPC1B, we edited the human Ramos B cell line using CRISPR/Cas9 to delete its expression (ARPC1B-KO cells) (Figure 1E). Like LCLs, control Ramos cells also spontaneously formed lamellipodia in suspension, and these structures were entirely dependent on ARPC1B (Figure 1F). This would suggest that ARPC1B is the major isoform that supports ARP2/3 complex–mediated F-actin branching in the steady state and that stimulation of the complex is ongoing, including in “resting” B cells.

### ARPC1B is required for WASP verprolin/cofilin/acidic (VCA) domain–stimulated F-actin nucleation.

**Figure 2:**
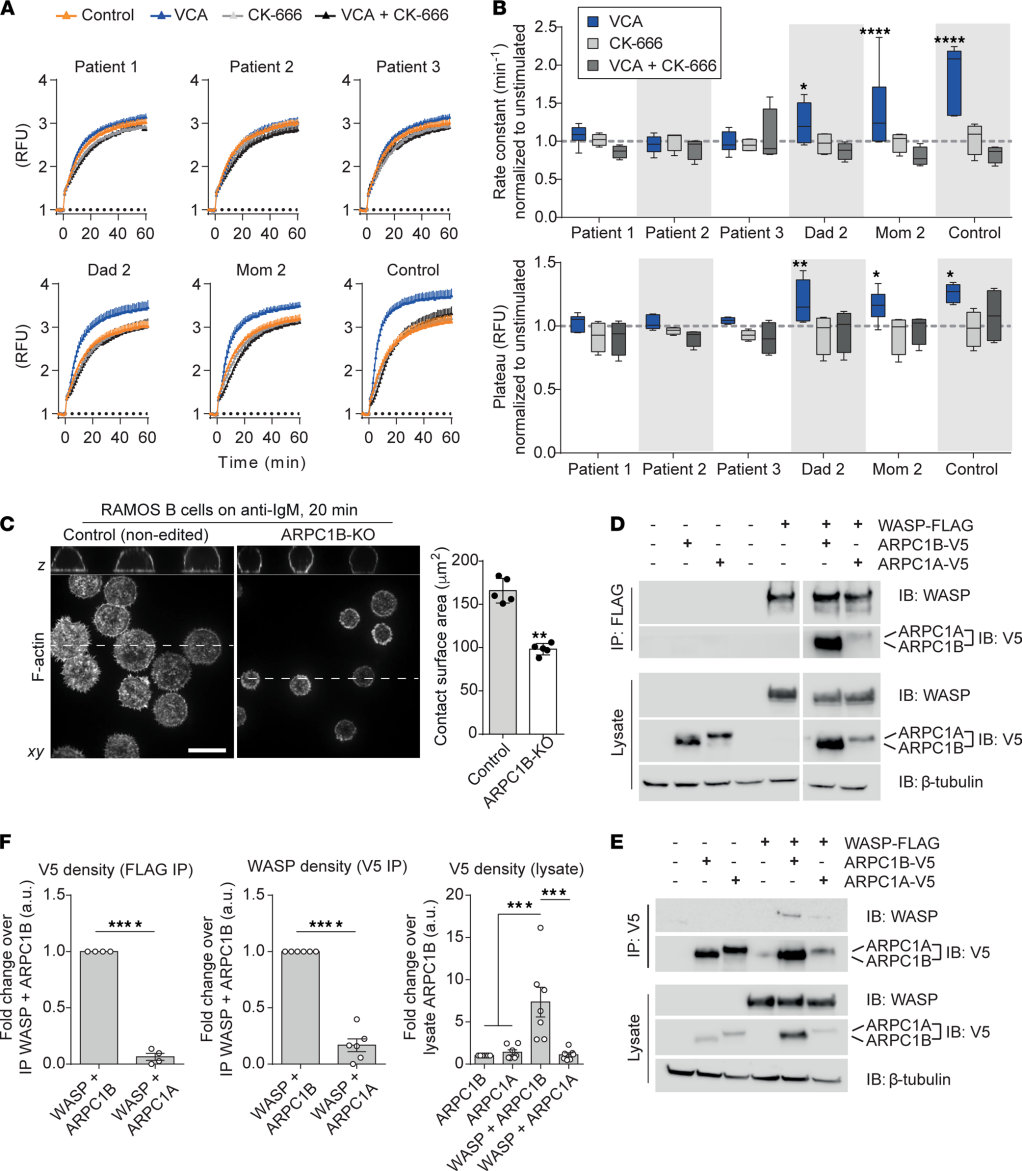
WASP binds to the ARPC1B subunit of the ARP2/3 complex to stimulate its nucleation. (**A**) Representative plots of actin polymerization assays in ARPC1B-deficient patient and control LCLs. Samples with pyrene-actin alone were recorded from t = –5 to 0 minutes to establish a baseline. Standardized whole cell lysates together with ATP and actin polymerization buffer ± VCA ± CK-666 (or vehicle controls) were added at t = 0 minutes and recorded every minute for 1 hour. Data are standardized as a fold change over the relative fluorescence units (RFU) at t = –5 minutes and represented as the mean ± SEM. (**B**) Data were analyzed as a plateau (maximal activity) followed by 1 phase association. The actin polymerization rate constants and activity maxima were calculated, normalized to the unstimulated sample within each sample, and represented as 2.5–97.5 percentile box plots; *n* > 4. For each data set, a 2-way ANOVA was performed followed by a Tukey’s multiple-comparison test; significance represents the comparison between VCA and VCA + CK-666 within the same sample. See also Supplemental Figure 2. (**C**) WT (control) and ARPC1B-KO Ramos B cells on anti-IgM–coated coverslips for 20 minutes, stained with Alexa Fluor 488–phalloidin (F-actin) (left). Scale bar: 10 μm. Contact surface area (μm^2^) was measured for 3–5 fields containing > 5 cells (right); *n* = 5. (**D** and **E**) Representative coimmunoprecipitation blots performed in HEK-293 cells. (**D**) Lysates from cells overexpressing full-length WT human WASP and ARPC1B or ARPC1A constructs were pulled down with FLAG beads (WASP); *n* = 4. (**E**) Representative blot showing samples immunoprecipitated using V5 beads (ARPC1B or ARPC1A); *n* = 6. (**F**) Densitometry analysis of ARPC1B and ARPC1A expression in cell lysates and pull-down proteins; *n* = 7. IP densitometry was compared using Student’s unpaired 2-tailed *t* test. Lysate densitometry was compared using a 1-way ANOVA with Tukey’s multiple-comparison test. Data are represented as the mean ± SEM. *P < 0.05; **P < 0.01; ***P < 0.001; ****P < 0.0001.

The stimulation of ARP2/3 complexes to nucleate F-actin branching is facilitated by membrane-targeted NPFs. When in an open, active conformation, these factors expose their VCA domains to then bring actin monomers out of solution and in close proximity to the ARP2 and ARP3 subunits of ARP2/3. Since the steps in F-actin branching are complex, we opted to first grossly determine the maximum contribution of VCA-stimulated ARP2/3 to the overall actin polymerization by performing pyrene-actin polymerization assays in the presence of B cell lysates derived from control or patient cells. We assessed the extent and rate of filament assembly in the lysates with or without the addition of a purified GST-VCA domain from WASP and found that the addition of the purified VCA domain to the lysates was sufficient to cause a more robust and rapid polymerization of actin as compared with lysates alone (compare orange and blue lines in Figure 2A). Inhibition of the ARP2/3 complex using a reversible and specific inhibitor CK-666 prevented any contribution from the recombinant WASP VCA domain in a dose-dependent manner (Figure 2, A and B, and Supplemental Figure 2, A and B). Importantly, VCA-stimulated actin polymerization was completely abolished in all ARPC1B-deficient patient cell lines (Figure 2B), suggesting that all of the VCA-stimulated ARP2/3 activity of the lysate required ARPC1B specifically.

NPFs are activated downstream of BCR signaling (24). Clustering of the BCR often occurs in a spatially regulated manner, where B cells encounter arrays of antigens displayed on the surface of presenting cells, resulting in their rapid spreading driven by F-actin branching (25, 26). We mimicked this response by seeding B cells onto planar arrays of anti-IgG to cross-link the BCR and assessed actin polymerization at the plane of contact with cross-linking antibodies. We found that LCL cells lacking ARPC1B were unable to polymerize actin on the planar surface, showing virtually no increases in their F-actin density at the sites of contact (Supplemental Figure 2C). Moreover, ARPC1B-KO Ramos cells were unable to spread on planar surfaces engaging their BCRs with anti-IgM (Figure 2C). Taken together, the loss of ARPC1B in the B cells caused weakening and dysregulation of both the steady-state cortical F-actin cytoskeleton and the loss of actin polymerization in response to BCR stimulation.

### WASP preferentially binds and stabilizes ARPC1B versus ARPC1A.

Because ARPC1B supported the ARP2/3-mediated processes of B cells, and since ARPC1B deficiency causes WAS-like disease, we investigated the role of WASP in the activation of ARPC1B- versus ARPC1A-complexes a priori. Indeed, WASP regulates B lymphocyte function, and its intrinsic loss in B cell populations causes autoimmunity (24, 27). The interaction between WASP and the ARP2/3 complex has also recently been shown to be in a 2:1 stoichiometry where each VCA domain has a distinct binding site: the ARP3/ARPC3 interface and ARP2/ARPC1 interface (28). As the ARPC1 subunit forms one of the VCA binding sites, and WASP VCA-domain stimulation of ARP2/3 varies with ARPC1 isoform, we hypothesized that ARPC1B may have a higher binding affinity for WASP as compared with the ARPC1A. To test this, we ectopically expressed tagged versions of the ARPC1 isoforms at equal amounts with or without a FLAG-tagged version of WASP in HEK293 cells to then immunoprecipitate WASP-FLAG. We found that WASP coimmunoprecipitated to a greater extent with ARPC1B as compared with ARPC1A with remarkable consistency (Figure 2, D and F) and that this finding could be reproduced in reverse experiments (Figure 2, E and F). Interestingly, the expression of WASP was sufficient to cause a robust increase in the stability of the ARPC1B subunit, as evidenced by its enhanced expression (Figure 2, D–F). Taken together, the binding of WASP to ARPC1B-containing ARP2/3 complexes and its nucleation of branched F-actin serves to stabilize ARPC1B protein expression, suggesting a coordinative function between WASP and ARPC1B-containing ARP2/3 complexes.

### ARPC1B regulates the B cell cortical actin cytoskeleton.

**Figure 3:**
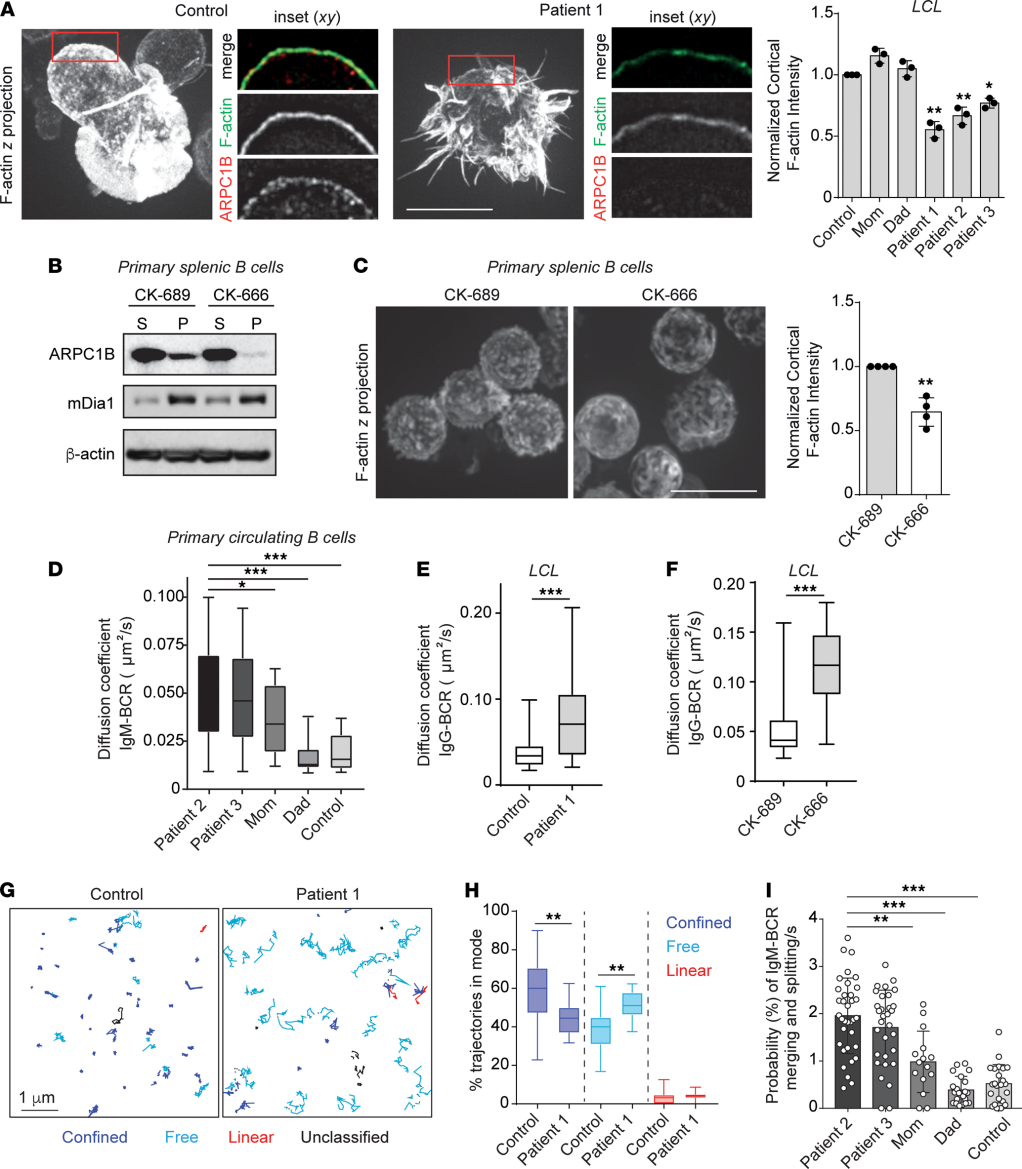
ARPC1B-containing ARP2/3 complexes in the cortex constrain the lateral diffusion and merging or splitting of the BCR. (**A**) LCLs fixed and stained for F-actin and ARPC1B in suspension. The cortical F-actin intensity was determined for control and patient cell lines by measuring the mean fluorescence for 3–5 regions of the cortex for > 30 cells; *n* = 3. Data are represented as the mean ± SEM. (**B**) Primary splenic B cells treated with CK-689 (control) or CK-666 for 1 hour were incubated in cytoskeleton stabilization buffer and fractionated to determine the cytoskeletal-associated proteins (pellet, P) from those in the cytosol (solution, S). (**C**) Primary splenic B cells were fixed and stained in suspension. Scale bars: 10 μm. The cortical F-actin intensity (right) was determined as in **A**. Data are represented as the mean ± SEM. (**D**–**I**) Individual BCRs within the plasma membrane were tracked over 10- to 20-second periods at 10 Hz in cells immobilized on anti–MHC-II–coated surfaces. (**D**) In primary B cells from ARPC1B family 2, median diffusion coefficients for IgM-BCRs was measured from > 25 cells and > 2500 trajectories. (**E** and **F**) The median diffusion coefficient of IgG-BCRs was calculated from control or patient LCLs and those treated with CK-666 or CK-689. (**G** and **H**) Representative trajectories of IgG-BCR in control and patient 1 LCLs color-coded using an MSS analysis for motion types. Confined tracks (blue), free tracks (cyan), linear tracks (red), and unclassified tracks (black) are shown from 1 representative cell (**G**) and quantified (**H**) for > 20 cells, > 5000 BCR trajectories; *n* = 3. Data were analyzed using Mann Whitney *U* tests. Scale bar: 1 μm. (**I**) Merging and splitting analysis of IgM-BCRs in primary B cells derived from the same experiments described in **D**. *P < 0.05; **P < 0.01; ***P < 0.001.

Given that ARPC1B deficiency leads to the production of autoantibodies, we hypothesized that the loss of functional ARP2/3 complexes may impact the threshold for B cell activation. Such a threshold can be governed by the cortical actin cytoskeleton that restrains BCR signaling (29). We found that ARPC1B complexes localized to the actin cortex of control LCL B cell lines and that lines derived from ARPC1B-deficient patients showed decreased and irregular cortical F-actin intensity (Figure 3A). We also found that ARPC1B was present in the F-actin fraction of resting primary B cells solubilized in a cytoskeleton stabilization buffer. Strikingly, when the B cells were treated with the ARP2/3 inhibitor CK-666, ARPC1B no longer associated with the cytoskeletal pellet (Figure 3B), and this pharmacological treatment also led to an irregular cortical F-actin appearance in the primary B cells (Figure 3C). The cortices of resting, ARP2/3-inhibited B cells showed thickened/bundled actin cables with large, nondiffraction limited gaps between them when compared with their control counterparts (Figure 3C). Interestingly, we found that the linear, processive nucleation promoting factor mDia1 remained in the cytoskeletal fraction when cells were treated with CK-666 (Figure 3B), suggesting that a balance between ARP2/3- and formin-mediated actin polymerization is important for maintenance of the resting B cell cortex.

### ARPC1B-containing ARP2/3 complexes branch F-actin networks that constrain the lateral diffusion of the BCR.

Tonic signaling in B cells is supported by metastable events involving the spontaneous homo- and heterotypic clustering of the BCR with itself and coreceptors. The submembrane actin cytoskeleton that furnishes the inner leaflet of the plasma membrane curtails these collisions by forming corrals or partitions that restrict the free diffusion of membrane-associated proteins and even lipids (30). Since the inhibition of ARP2/3 disrupted the cortical cytoskeleton, we suspected that ARPC1B may play an important role in controlling the like collisions of the BCR by forming membrane corrals (19) or other submembrane domains (31–33).

To test this, we labeled and tracked individual IgM-BCRs in primary circulating human B cells isolated from 2 of the ARPC1B-deficient patients and 3 controls. Of note, patient 1 with the most severe disease had received hematopoietic stem cell transplantation by the time of these experiments (Figure 1). The primary B cells were seeded onto anti–MHC-II surfaces in order to monitor the diffusion of BCRs in a 2-dimensional plane while minimizing stimulation of the cells as done previously (19). By measuring the diffusion coefficient of single BCRs, we found that ARPC1B-deficient patients showed a 3- to 4-fold increase in the diffusion of IgM-BCRs compared with control counterparts (Figure 3D). Such increases in diffusion were not limited to IgM. By analyzing BCR diffusion in patient and control LCL lines, we found that IgG-BCRs also showed an increase in diffusion from ~0.03 to 0.07 μm^2^/s (Figure 3E). Increases in the diffusion of the BCR in the absence of ARPC1B was likely attributed to the loss of functional ARP2/3 complexes, as demonstrated for other cell lines (34) and as recapitulated in LCL cells acutely treated with CK-666, which showed similar increases in BCR diffusion (Figure 3F).

To further examine the diffusion behavior of the BCR in patient cells, we used a moment scaling spectrum (MSS) analysis in order to categorize receptor trajectories as having undergone motion that was confined, free, or linear (Figure 3, G and H) (35). This analysis revealed that only a small portion of the IgG-BCRs underwent linear motion, suggesting that their lateral diffusion is not driven by motor-based movement along cytoskeletal polymers. Instead, we found that the majority of receptors underwent random motion that was either confined or freely diffusing. When we compared ARPC1B-deficient B cells to controls, we found IgG-BCRs were more likely to undergo free rather than confined motion in the absence of ARPC1B (Figure 3, G and H, and Supplemental Video 1). For receptors that were determined to be confined, the area of confinement was similar between control and ARPC1B-deficient cells (Supplemental Figure 3). This would suggest that, while ARPC1B may contribute to corralling the BCR, the corrals that remain in ARPC1B-deficient cells are indistinguishable from control cells. Alternatively, or in addition to this effect, ARPC1B may drive the active clustering of the receptors.

Ultimately, the like-collisions between BCRs are an important parameter for generating tonic signals, events that increase with more freely diffusing receptors (19, 29). We therefore measured merging and splitting events that lasted more than 5 time frames (0.5 second), which could be deciphered by algorithms that detect a doubling (or halving) of the spot intensity. This represents receptors coming within the diffraction limit of each other and maintaining the same pattern of diffusion, likely a consequence of metastable nanoclustering (36). We analyzed primary B cells to measure these merging and splitting events and found that ARPC1B-deficient B cells showed more metastable clustering of the IgM-BCRs as compared with controls (Figure 3I). Taken together, these data support the idea that ARP2/3 complexes containing ARPC1B restrict the diffusion of BCRs and limit their spontaneous, homotypic coalescence in the plasma membrane.

### ARPC1B deficiency leads to increased calcium and PI3 kinase signaling in B cells.

**Figure 4:**
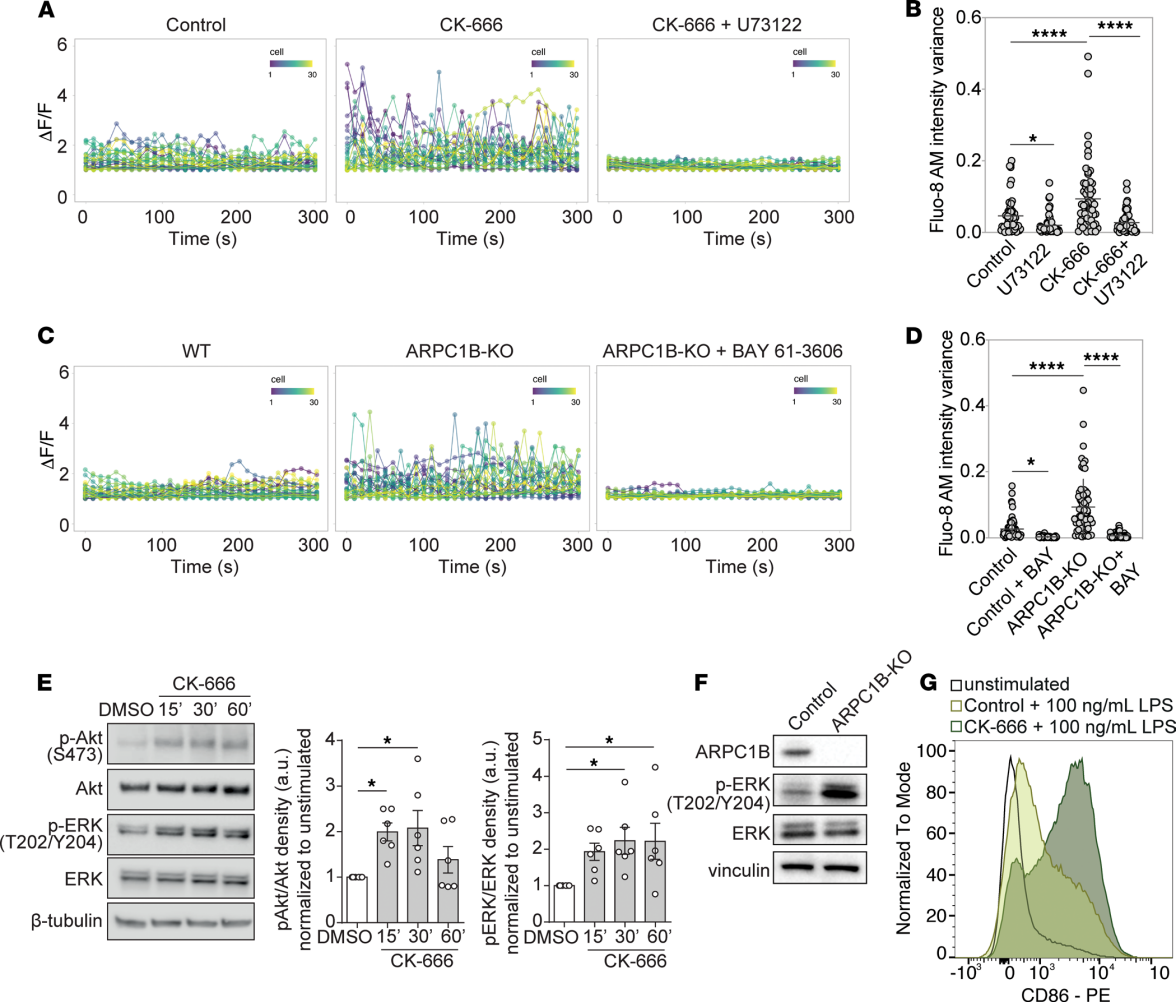
The loss of cortical ARPC1B-containing ARP2/3 complexes increases spontaneous, oscillatory calcium signaling, phospho-Akt, and CD86 expression. (**A** and **B**) Control (CK-689) or CK-666–treated Ramos B cells loaded with the Fluo-8 calcium sensor were imaged every 10 seconds for 5 minutes; they were then treated with the PLC inhibitor U73122 and imaged for another 5 minutes (middle panel). Ensemble traces are shown in **A**. (**B**) The variance (deviation from the mean) was determined as a measure of [Ca^2+^]_cytosol_ flux. Greater than 50 cells were used; *n* = 3. (**C** and **D**) WT and ARPC1B-KO Ramos cells imaged and quantified as in **A**. Cells were treated with the SYK inhibitor BAY 61-3606 for 10 minutes and imaged for another 5 minutes. (**D**) The variance was determined as a measure of [Ca^2+^]_cytosol_ flux. Greater than 50 cells were used; *n* = 3. (**E**) Ramos cells blotted for AKT and ERK phosphorylation at indicated times after addition of CK-666. Results were assessed using a 1-way ANOVA and Dunnett’s test comparing all groups to the DMSO control; *n* = 6. (**F**) Unstimulated WT and ARPC1B-KO Ramos cells blotted for ERK phosphorylation. (**G**) Primary B cells given Control (CK-689) or CK-666 overnight together with 100 ng/mL LPS. Representative surface CD86 by FACS is shown; *n* = 3. *P < 0.05; ****P < 0.0001.

Signaling from the BCR is essential for the survival of B cells in the periphery, yet these events are minute and transient, making them difficult to capture experimentally. We nevertheless reasoned that since BCR activation leads to the release of Ca^2+^ from ER stores (by producing IP_3_ [inositol triphosphate] through the PLC-mediated cleavage of PtdIns[4,5]P_2_ [phosphatidylinositol 4,5-bisphosphate]), we would be able to derive quantitative assessments of tonic, transient signaling events with highly sensitive approaches for measuring cytosolic [Ca^2+^] in single cells over time. To this end, we opted to use Fluo-8 AM, a cell-permeant Ca^2+^ binding dye that detects small elevations of [Ca^2+^]_cytosol_, normally kept within the nanomolar range by Ca^2+^ ATPases. Ramos B cells were briefly (5 minutes) loaded with Fluo-8 AM at concentrations that minimized the chelation of Ca^2+^ and toxicity to the cells. We subsequently recorded the cells at regular, 10-second intervals to monitor cytosolic Ca^2+^ fluxes. Example traces of the Ca^2+^ signal over time are shown in Figure 4A, and an example video is shown in Supplemental Video 2. From these recordings, we detected only minor fluxes in the [Ca^2+^]_cytosol_ in resting Ramos B cells, graphically depicted as the variance (deviation from the mean) of the signal for cells over a 5-minute recording (Figure 4B). The small variance was ostensibly driven by phospholipases and IP_3_ production. Treating the same cells with a PLC/PLA2 inhibitor U73122 quieted the [Ca^2+^]_cytosol_ signal almost instantaneously (Figure 4, A and B).

Using the same approach, we monitored the [Ca^2+^]_cytosol_ flux over time in cells treated with CK-666. Remarkably, after 20 minutes of ARP2/3 inhibition, we found that the B cells began to show steep oscillations of the [Ca^2+^]_cytosol_ signal that were inhibited by the phospholipase inhibitor to the same extent as control cells (Figure 4, A and B, and Supplemental Video 2). The variance in the [Ca^2+^]_cytosol_ signal changed 2-fold upon the inhibition of ARP2/3 (Figure 4B).

To determine if the elevated variance in the [Ca^2+^]_cytosol_ signals depended on ARPC1B specifically, we loaded ARPC1B-KO Ramos B cells with Fluo-8 AM. When compared with their control counterparts, the ARPC1B-KO B cells indeed showed more frequent and robust oscillations of [Ca^2+^]_cytosol_ (Figure 4, C and D, and Supplemental Video 3). Moreover, the Ca^2+^ oscillations ostensibly required BCR signaling, since these were inhibited upon targeting the activity of the tyrosine kinase, SYK, with BAY 61-3606 (Figure 4, C and D). Thus, tonic release of Ca^2+^ from the ER via the activation of SYK and the production of IP_3_ is elevated in the absence of ARPC1B-containing ARP2/3 complexes.

The treatment of B cells with actin-depolymerizing compounds also causes an elevated phosphorylation of Akt and ERK (19). To discern if some of this effect is dependent on ARP2/3, we treated primary B cells or the Ramos human B cell line with CK-666. Indeed, inhibiting ARP2/3 branching led to increased p-Akt and p-ERK in a manner that depended on SYK tyrosine kinase activity, suggestive of being downstream of the BCR (Figure 4E and Supplemental Figure 3). ARPC1B-KO Ramos B cells also showed higher p-ERK signals compared with WT (Figure 4F). These data are consistent with a role for ARPC1B in curtailing tonic signaling in B cells.

Upon the engagement of TLRs, the B cell cortex is remodelled via F-actin severing pathways. This represents a mechanism of receptor crosstalk, whereby TLRs decrease the threshold for BCR-mediated B cell activation (29, 37). We therefore tested if the loss of ARP2/3 branching activity could lower the threshold for B cell responses to LPS, a TLR4 ligand. Targeting ARP2/3 complexes with CK-666 indeed augmented the phosphorylation of AKT and ERK in response to TLR4 engagement (Supplemental Figure 3). The inhibition of ARP2/3 also lowered the threshold for LPS-induced activation of primary B cells, as measured by their surface expression of CD86, a costimulatory ligand for T cells (Figure 4G). Taken together, these results implicate ARP2/3 complexes in curtailing tonic signaling from the BCR and, thereby, constraining the crosstalk to the BCR from microbial associated molecular patterns.

### ARPC1B is required for the formation of podosomes in macrophages.

**Figure 5:**
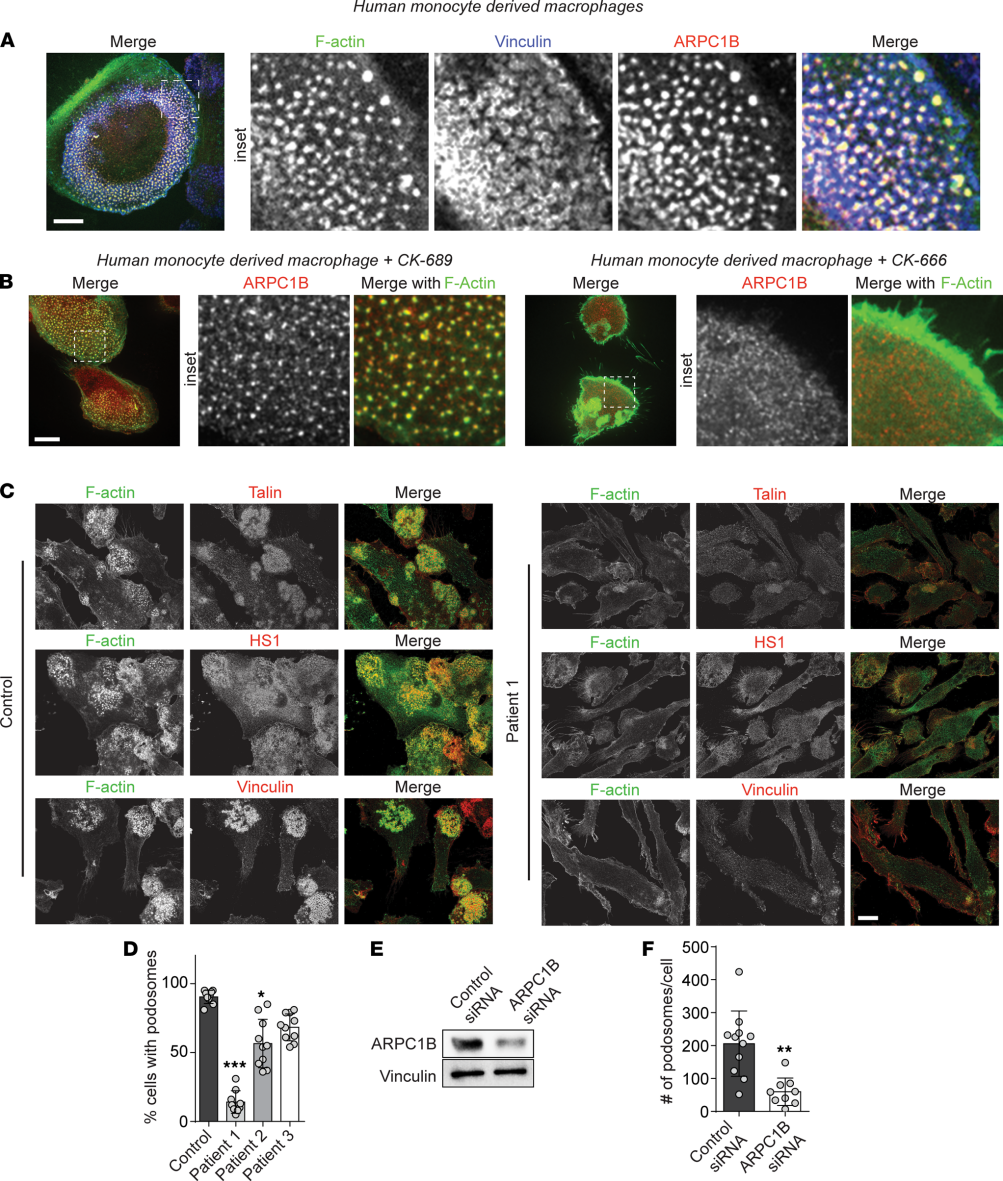
ARPC1B is critical for ARP2/3-dependent podosome formation and function. (**A**) Primary human monocyte–derived macrophages (hMDM) fixed and stained for F-actin, Vinculin, and ARPC1B on cover glass. (**B**) hMDM treated with CK-689 (control) or the ARP2/3 inhibitor CK-666 stained for ARPC1B and F-actin. (**C**) Healthy control and patient MDM fixed and stained as in **A** for Talin, HS-1, and Vinculin. (**D**) The percentage of cells that form podosomes imaged as in **A** and quantified for > 25 cells; *n* > 2. Data are shown as mean ± SD. (**E**) Representative Western blot for ARPC1B in control and silenced primary macrophages. (**F**) The number of podosomes per cell in control siRNA versus ARPC1B siRNA–treated hMDM. Data are shown as mean ± SD. Scale bars: 10 μm. *P < 0.05; **P < 0.01; ***P < 0.001.

The branching of actin networks by WASP and ARP2/3 is critical for numerous immune cell functions, including for podosomes, small (~1 μm) adhesive structures unique to myeloid cells that contain dense F-actin cores surrounded by a ring of active integrins (38). The polymerization of actin in the podosome core is especially dependent on the activation of WASP; the adhesive structures are absent in WAS patients (39). To determine if it is ARPC1B-containing ARP2/3 complexes that feature in a discernibly WASP-mediated cytoskeletal process, we took advantage of this established podosomal biology and differentiated primary circulating human monocytes into macrophages to investigate the localization of ARPC1B vis-à-vis podosomes. We noted that a majority of the primary human macrophages formed podosomes, as marked by rings of Vinculin, an integrin-associated protein, that surrounded F-actin cores (Figure 5A). Remarkably, the podosome cores were also highly enriched for ARPC1B, which colocalized with the F-actin (Figure 5, A and B). Disrupting the podosomes with CK-666 dislodged any association between ARPC1B and the plasma membrane (Figure 5B). Importantly, we found that ARPC1B-deficient macrophages showed an impairment or virtually no podosome structures as measured by Vinculin and Talin, another integrin-associated protein, and the ARP2/3-associated protein HS-1 (Figure 5C). The extent of podosome loss in primary patient macrophages correlated with their expression of ARPC1B (Figure 5, B and D). Silencing the expression of ARPC1B using RNA interference similarly decreased podosome assembly (Figure 5, E and F), demonstrating that even the acute loss of ARPC1B led to the inability for the cells to form podosomes. Taken together, ARPC1B deficiency is marked by the loss of podosomes in primary macrophages, phenocopying WAS macrophages.

### ARPC1B is required for matrix degradation but not phagocytosis.

**Figure 6:**
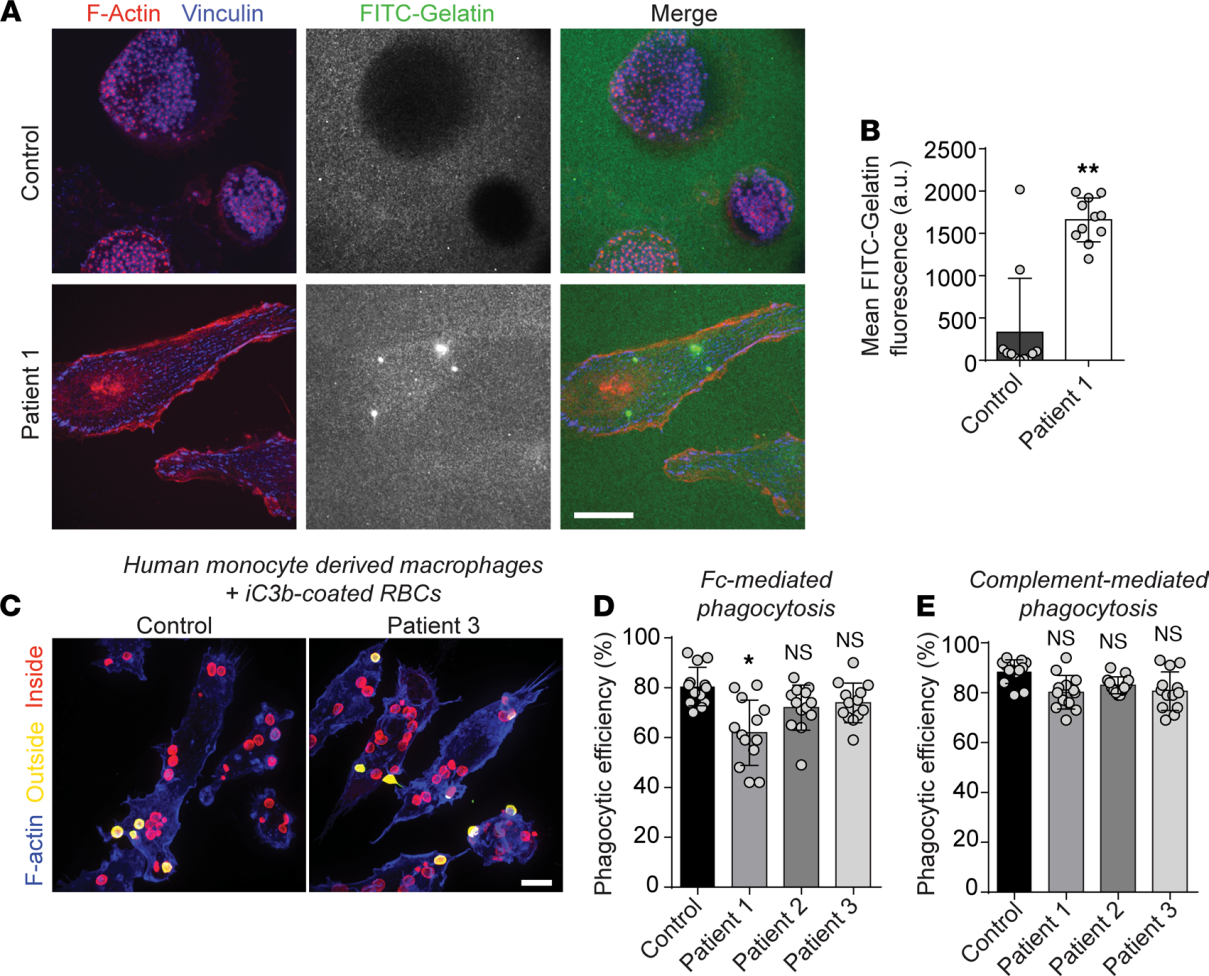
ARPC1B deficiency leads to impaired matrix degradation but only minor defects in phagocytosis. (**A**) Primary human monocyte–derived macrophages (hMDM) were seeded onto FITC-gelatin coated coverslips for 2–3 hours and stained as indicated. (**B**) The mean FITC signal underneath the cells as in **A** was quantified for > 10 fields and > 30 cells. (**C**–**E**) hMDMs challenged with red cells opsonized with anti–RBC IgG or C5-deficient serum (C3b) for 10 minutes were fixed and stained for outside and inside opsonin. Representative images (**C**) and phagocytic efficiency (**D** and **E**) as determined by the number of particles inside divided by the total number of particles. Data are shown as the mean of >10 fields and > 30 cells. Scale bar: 20 μm. *P < 0.05; **P < 0.01.

To understand if defects in the innate immune compartment contributed to the clinical features of ARPC1B deficiency, we assessed the ability for the patient macrophages to remodel extracellular matrix and phagocytose particles. In addition to their role in adhesion, podosomes are (a) sites of transmembrane matrix metalloproteinases (MMP) recruitment and retention that mediates the local degradation of the apposing ECM (38) and (b) features in phagocytosis (40); these are both functions that could impact disease. To determine if ARPC1B-deficient macrophages were able to degrade their substrata, we seeded macrophages derived from ARPC1B-deficient patients or control donors onto fluorescently labeled gelatin. Under such conditions, macrophages expressing ARPC1B degraded the gelatin, while those from ARPC1B-deficient patients did not (Figure 6, A and B). Despite the defects in matrix degradation and in podosome formation, upon challenging control and patient primary macrophages with various phagocytic targets, we found only minor decreases in Fc- and complement-mediated phagocytosis (Figure 6, C–E), which had been previously been described as ARP2/3-dependent processes (41, 42). We thus concluded that either the remnant expression of ARPC1B or the upregulated ARPC1A seemed to suffice for phagocytic efficiency and that alterations in phagocytosis are unlikely to explain the immune defects associated with ARPC1B deficiency.

## Discussion

Tonic signaling from immunoreceptors in resting cells is regulated by their transient associations with the submembrane cytoskeleton. We found that ARP2/3 complexes stimulated by WASP in B cells and macrophages were made with the ARPC1B isoform and that the resultant branched actin polymers controlled the cell cortex and podosomes, respectively. Patients with ARPC1B deficiency showed elevated ARPC1A protein expression, but this was insufficient to compensate for the loss of ARPC1B in most processes, except for Fc- and complement-mediated phagocytosis. The absolute concentrations of ARPC1A in healthy control and ARPC1B-deficient patient cells remains unknown, as is the source of ARPC1A upregulation; it may involve epigenetic changes in progenitor populations or the stabilization of the ARPC1A protein itself. When expressed at comparable levels, ARPC1B was stabilized in the presence of WASP, but ARPC1A was not, suggesting that ARPC1 isoforms are regulated by their incorporation into branched F-actin networks. ARPC1A may therefore remain expressed at low levels in ARPC1B-deficient cells but become partially stabilized compared with healthy controls by NPFs other than WASP.

This raises the possibility that different NPFs recognize ARP2/3 complexes made up of different isoform combinations. Once assembled, the stability of ARP2/3-branched F-actin networks has also recently been shown to depend on the isoforms of ARP2/3 components ARPC1, ARPC5, and ARP3 (10, 43). ARP2/3 isocomplexes composed of ARPC1B and ARPC5L are more stable than those containing ARPC1A and ARPC5, owed to their interaction with cortactin, which opposes debraching by coronin (10). ARP3B-containing complexes, on the other hand, form branch points of a lower stability than those with ARP3, as the former is oxidized by MICAL2, which is recruited by coronin 1C (43). Taken together, ARP2/3 branching has recently evolved to include a family of complexes that have different properties, including their stabilization by cortactin and their stimulation by NPF partners. The coexpression of ARPC1B and WASP in hematopoietic cells and the comparable features of ARPC1B deficiency and WAS would suggest that the 2 proteins indeed function in a concerted fashion. Whether or not N-WASP, another NPF that shares 50% sequence homology with WASP and is expressed in B cells (27), can also stabilize ARPC1B is not known.

WAS involves intrinsic B cell defects that lead to autoimmunity (5, 44). WAS^–/–^ patients and mice have B cell compartments enriched for cells with self-reactive, low-affinity surface BCRs. Kolhatkar et al. demonstrated that this is not the result of aberrant negative selection but that the transitional B cell compartment receives overzealous signals from the BCR that lead to enhanced proliferation in the absence of functional WASP (44). Here, even modest alterations in the surface signals are sufficient to alter B cell tolerance and support a role for transitional B cells in autoimmune disorders like WAS (44). In fact, in a hematological analysis of patients with ARPC1B deficiency, we found high absolute counts for transitional B cells of 123 × 10^6^/L and 101 × 10^6^/L for patients 2 and 3, respectively, well above the reference range and that of heathy age-matched controls that were 41 × 10^6^/L. At the time of this analysis, patient 1 had already received hematopoietic stem cell transplant. These counts nevertheless support the hypothesis by Kolhatkar et al. (44) that transitional B cell numbers are elevated without regulation of their cortical cytoskeleton and tonic signals by ARPC1B/WASP. Whether or not ARPC1B deficiency also increases responses to soluble or particulate BCR ligands remains unknown; however, the role of F-actin branching in B cell responses is bound to be complex (see below).

Other actin regulatory genes expressed in hematopoietic cells are mutated in autoimmune disorders beyond WAS and ARPC1B deficiency. Recently, the hematopoietic-specific WASP-family verprolin-homologous protein (WAVE) regulatory complex (WRC) component HEM1 was found to be essential in B cell development (45). Like WAS and ARPC1B deficiency, patients with HEM1 deficiency have severe immune dysregulation. While the loss of HEM1 also reduces levels of F-actin, HEM1^–/–^ B cells have reduced tonic BCR signals and decreased numbers of transitional B cells. As a result of weakened BCR signal strength, B cell fate was skewed toward autoreactive B cells. The WRC is an NPF for the ARP2/3 complex, and since ARPC1B is the only isoform expressed in B cells, these complexes presumably contain ARPC1B. In the absence of HEM1, and due to a reduced stability of the WRC, it is conceivable that WASP-mediated activation of the ARP2/3 complex is elevated. The findings of this report together with that of Kolhatkar et al. and Salzer et al. (44, 45) would indeed suggest that WASP and WAVE have diametrically opposed regulation on the strength of tonic BCR signals. Whether the NPFs act at the cortex vis-à-vis the BCR, CD19, Siglecs, and phosphatases that regulate tonic BCR signaling events will be of great future interest.

Several other proteins involved in the regulation of the actin cytoskeleton, including DOCK2 (46), DOCK8 (47), activators of Rac GTPases, and the WRC, have also been shown to be critical in B cell responses, but these are involved in migration and immune synapse formation. In this regard, the role of F-actin branching by WAVE, WASP, and neural-WASP (N-WASP) may have entirely different roles that regulate BCR signal strength when engaged by cognate antigens on planar surfaces. First, ARP2/3-branching and cell spreading supports increased surface contact with the antigen presenting cell (APC) and facilitates more antigen gathering. This is expected to enhance signaling from the BCR during the rapid spreading phase (26, 34). Second, antigens are extracted from the APC surface and endocytosed (48) — processes that will increase the presentation of antigen by the B cell and elicit T cell help. Finally, antigen-loaded MHC-II needs to be trafficked to the surface of B cells to have an effect. The trafficking of peptide–MHC-II has been shown to require extensive remodeling of the endolysosome compartment (49). Such remodeling may also be driven by actin polymerization and ARP2/3 nucleation via yet another NPF, WASH (50). A role for ARPC1B in all of these steps seems likely.

We also found that ARPC1B was required for signals emanated from podosomes in macrophages, sites of integrin, and tyrosine kinase activation. Though low levels of ARPC1B expression in patients was sufficient to maintain podosome structures, complete loss of ARPC1B led to no podosomes being formed and ablated the ability for macrophages to remodel gelatin matrices. The immunoreceptors/ITAMs (immunoreceptor tyrosine activation motif) involved in podosome signaling are not yet determined; however, the ITAM-containing adaptor DAP12 (DNAX activating protein of 12 kDa) and the common FcRγ chain are required for steady-state signaling from integrins in macrophages (51). Patients with ARPC1B deficiency have developmental defects and thrombocytopenia. The low level of platelets in the patients could result from the loss of podosome-like structures formed by the proplatelets of megakaryocytes in the BM. A deeper understanding for the signaling that assembles these structures, guilding the proplatelet to the vasculature, is imperative to further appreciate ARPC1B deficiency.

In immune cells, the tonic signals that support mitogenesis and survival must be integrated over time. While tonic signaling from the BCR is unique, all immune cells receive signals from their microenvironment in the absence of infection of inflammation. For example, in sterile environments where selection takes place, thresholds are set for how lymphocytes gauge events that should trigger proliferation in the periphery. In settings where sterility is breached, TLR signals lower the threshold for activation and, on their own, stimulate B cell expansion and blasting. Certain populations of innate-like B lymphocytes (e.g., marginal zone B cells) are more susceptible to nonspecific expansion. A role for ARPC1B in marginal zone B cells and the production of natural antibodies will be of particular interest. Generally, tonic signaling is challenging to study since the events are small; for example, only minute amounts of pAKT, pERK, and pCD79a are observed in resting B cells even when ARP2/3 is inhibited compared with engaging the BCR with crosslinking antibodies. However, small differences compounded over time are expected to influence disease and lead to autoimmunity, as is the case in ARPC1B deficiency. As we begin to understand more about the thresholds set for activation and the regulation for tonic, steady-state signals, it is becoming clear that new tools, including reporter animals, will be necessary to quantify these events over time (22, 34).

This work provides mechanisms that lead to the disease pathology in ARPC1B deficiency and improves our understanding of the ARP2/3 complex composition and regulation in hematopoietic cells. Further studies will be critical in determining whether the B lymphocytes should be targeted in patients with ARPC1B deficiency.

## Methods

### Cell isolation and culture

#### Primary B cell isolation.

PBMCs were isolated using SepMate tubes (Stemcell Technologies) with Ficoll-Paque PLUS density gradient media (GE Life Sciences) and selected for B cells using the EasySep human B cell isolation kit (Stemcell Technologies) as per the manufacturer’s instructions. Control B cells were derived from an unrelated healthy male human.

#### Primary human macrophages.

Primary human monocytes were isolated from heparinized blood of donors. Peripheral blood mononuclear cells were first isolated using Lympholyte-H (Cedarlane), resuspended in RPMI, and seeded onto tissue culture-treated plastic dishes for 20 minutes to select adherent cells. Nonadherent cells were removed by washing with RPMI medium (Wisent Bioproducts, catalog 350-000-CL). Adherent cells were then incubated in RPMI with L-glutamine containing 10% heat-inactivated serum, 100 U/mL penicillin, 100 mg/mL streptomycin, and 10 ng/mL hM-CSF (PeproTech, catalog 300-25) for 7 days.

#### Cell lines.

EBV-transformed lymphoblasts were established from patient B cells following standard protocols by The Centre for Applied Genomics at SickKids. LCLs were maintained in RPMI 1640 medium supplemented with 15% FBS (Gibco, catalog 12483020) and 2% antibiotic/antimycotic solution (Wisent Bioproducts, catalog 450-115-EL). Control LCLs were derived from an unrelated male with a noninflammatory phenotype and no known damaging mutations. HEK293 cells (ATCC) were maintained in DMEM medium (Wisent Bioproducts, catalog 319-007-CL) with 10% FBS and 1% antibiotic/antimycotic. Ramos cells were a gift from Michael Gold (University of British Columbia, Vancouver, Canada) and maintained in RPMI 1640 medium supplemented with 10% FBS and 1% antibiotic/antimycotic. ARPC1B-KO Ramos cells were generated using a p01 U6-gRNA:CMV-Cas9-2a-tGFP vector from MilliporeSigma containing MISSION gRNA HSPD0000060019. Cells were nucleofected using a Neon Electroporation System (Thermo Fisher Scientific) and sorted singly by FACS after 24 hours. Clones were expanded and used 3–4 weeks after verifying KO using anti-ARPC1B antibodies.

### Actin polymerization assay

The pyrene actin assay protocol was modified from the Actin Polymerization Kit from Cytoskeleton Inc. Cells were collected in 20 mM Tris-HCl (pH 7.5) with 20 mM NaCl. Actin buffer (67 μL of 5 mM Tris-HCl [pH 8.0], 0.2 mM CaCl_2_, 0.4 mg/mL pyrene-labeled rabbit muscle actin, 2 μM ATP) was added to a black polystyrene flat-bottom 96-well plate (Greiner) and read every minutes for 5 minutes at room temperature (RT) using a SpectraMax Gemini EM fluorescence microplate reader (Molecular Devices) with SoftMax Pro software (Molecular Devices). Immediately, the plate was removed, and the following was added: 5 μg recombinant GST-VCA (Cytoskeleton Inc.) or 5 μL H_2_O, 800 μM CK-666 (MilliporeSigma) or 5 μL DMSO, cell lysates (36.4 μg in 33 μL), and 10 μL polymerization buffer (final concentration of 50 mM KCl, 2 mM MgCl_2_, 5 mM guanidine carbonate, 1 mM ATP). The reaction was read every minute for 60 minutes at RT. Data from each well were normalized to the first reading (i.e., RFU = 0 at t = –5 minutes).

### Immunoblotting

Cells were collected in lysis buffer (150 mM NaCl, 50 mM HEPES, 1% Triton-X 100, 10% glycerol, 1.5 mM MgCl_2_, 1.0 mM EDTA, 1 mM NaF, 0.2 mM Na_3_VO_4_, 1 mM PMSF; all from MilliporeSigma), and 30 μg of protein with Laemmli buffer was loaded onto a commercial 4%–20% gradient gel (Bio-Rad). The gel was transferred onto a nitrocellulose membrane using the Trans-Blot Turbo Transfer System (Bio-Rad). Membranes were blocked for 1 hour at RT in blocking buffer (5% skim milk in PBST). Primary antibodies were incubated for 1 hour at RT or overnight at 4°C in blocking buffer: anti-AKT (1:1000; Cell Signaling Technology, 9272), anti–phospho-AKT (1:1000; Cell Signaling Technology, 9271), anti-ARPC1A (1:1000; MilliporeSigma, HPA004334), anti-ARPC1B (1:1000; MilliporeSigma, HPA004832), anti-ARPC5 (1:1000; Abcam, ab118459), anti–β-tubulin (1:3000; Abgent, AP52742), anti-ERK (1:1000; Cell Signaling Technology, 4695), anti–phospho-ERK (1:1000; Cell Signaling Technology, 9101), anti-V5 (1:1000; Cell Signaling Technnology, 13202); and anti-WASP (1:1000; Cell Signaling Technology, 4860). Membranes were washed 3 times, for 5 minutes each time, with PBST; incubated for 1 hour at RT with HRP-conjugated goat anti-mouse (1:2000; Jackson ImmunoResearch, 115-005-003) or anti-rabbit IgG (Jackson ImmunoResearch, 111-005-003) in blocking buffer; washed 3 times; developed using Luminata Crescendo Western HRP substrate (EMD Millipore) or Clarity Max Western ECL Substrate (Bio-Rad); acquired with Odyssey Fc Imaging System (LI-COR Biosciences); and quantified with Image Studio software.

For coimmunoprecipitation studies, 2 mg of HEK293 cell lysate was sonicated and rotated overnight with 25 μL anti-FLAG M2 Affinity Gel beads (MilliporeSigma) or anti-V5 Agarose Affinity Gel beads (MilliporeSigma). Beads were washed 4 times with complete lysis buffer, heated at 95°C in Laemmli buffer for 10 minutes, and then run on a gel as described above.

### Single-particle tracking (SPT) of the BCR and merge-split analysis

Labeling and tracking of the BCR was done as previously described (29). Briefly, to label single membrane IgM (mIgM) or mIgG molecules, B cells were resuspended to 1 × 10^7^ cells/mL, blocked with 5% rat serum for 10 minutes, and subsequently incubated in HBSS for 5 minutes with 1 ng/mL Cy3 anti-IgM or anti-IgG Fab fragments (Jackson ImmunoResearch Laboratories, catalog 109-167-003 and 109-167-003). For merge-split analysis, Cy3-labeled anti-IgM Fab fragments were used at 5 ng/mL. All labeling steps were carried out at 4°C to minimize BCR internalization. After labeling, the cells were washed and warmed to 37°C before imaging.

B cells were immobilized for imaging; they were allowed to attach for 5 minutes to coverslips that had been coated with 1 μg/mL of the M5/114 anti–MHC-II monoclonal antibody (eBioscience, Clone M5/114.15.2 catalog 13-5321-82). In some experiments, B cells were pretreated with CK-666 (MilliporeSigma) for 30 minutes. Live-cell imaging was performed using a Zeiss Axiovert 200 epifluorescence microscope equipped with a 100× oil-immersion objective (NA 1.45), a custom-made 2.4× lens, and an Exfo X-Cite 120 light source. A Hamamatsu 9100-13 deep-cooled EM-CCD camera was used for recording, and Volocity software was used for image acquisition. Images were acquired continuously at 10 frames per second for 10–20 seconds.

To track single BCRs, Gaussian kernels were fit to local maxima intensities that were detected by imaging in order to determine particle positions. Particles were tracked using a 2-step algorithm to generate complete trajectories by closing gaps and capturing merging and splitting events. Generated tracks ended as a result of particle disappearance or the merging of particles. Tracks lasting at least 5 frames were retained for trajectory analysis.

BCR diffusion types were determined using a MSS analysis of particle displacements as previously described (29, 35). Particle diffusion coefficients were calculated from the MSS analysis. The confinement dimension for confined and linear trajectories was derived via eigenvalue decomposition of the variance-covariance matrix of particle positions along each trajectory as done by Jaqaman et al. (35).

### Microscopy and image analysis

Imaging was performed using a Quorum spinning disc mounted on a Zeiss Axiovert 200M microscope, using 63× or 25× objectives and a back-thinned EM-CCD camera (C9100-13, Hamamatsu), or it was performed using a Leica SP8 Lightning Confocal/STED using a 100×/1.4 STED objective. Acquisitions were controlled by the Volocity software (Perkin-Elmer) or Leica microsystems imaging software, exported, analyzed and quantified using Volocity or Fiji (NIH) software.

### Calcium flux

Prior to live cell imaging, cells were loaded with 0.5 μM of Fluo8-AM (Abcam) for 5 minutes in Tyrode’s buffer (140 mM NaCl, 10 mM glucose, 5 mM KCl, 2 mM CaCl_2_, 1 mM MgCl_2_, 10 mM HEPES [pH 7.4]). Cells were subsequently washed and imaged in Tyrode’s buffer at 6 frames per minute for 5–10 minutes. Using Fiji to analyze the video recordings, ROIs were generated to determine the average fluorescence signal over time, and the average variance (deviation from the mean) was determined per cell and graphed. Rare dead cells, as determined by their high calcium signal, were not included in the analysis.

### Phagocytosis

Macrophages were adhered to uncoated 18 mm coverglass for 24–48 hours. In all cases, particle binding and phagocytosis of targets was done as previously described (42, 52). Briefly, sheep erythrocytes (MP Biomedicals, catalog 55876) were opsonized with rabbit anti-sheep RBC IgG (MP Bio, catalog 55806) or with C5-deficient serum (MilliporeSigma, catalog 234405). All particles were added to the macrophages for 20 minutes at 37°C before fixation with 4% paraformaldehyde.

### Immunostaining

Cells were fixed with 4% PFA (Electron Microscopy Sciences) for 20 minutes at RT. Cells were then permeabilized with 0.5% Triton X-100 in PBS for 5 minutes, blocked with 2% BSA in PBS, and stained with the indicated antibodies in blocking solution (refer to Additional reagents). After staining, coverslips were mounted onto glass slides using ProLong Diamond mounting medium (Invitrogen). Antibodies used for immunofluorescence are described in reagents.

### Flow cytometry

Immunophenotyping of B cell subsets in the periphery was done as previously described (53). Additional antibodies used for FACS are from BioLegend, including anti–IgM-APC (catalog 406509), –CD80-PE (catalog 104711), and –CD86-Cy7-APC (catalog 105029).

### Additional reagents

For Western blotting analysis, primary B cells, macrophages, or B cell lines were lysed in RIPA lysis buffer (MilliporeSigma, R0278) containing protease inhibitors (Thermo Fisher Scientific, A32963) and phosphatase inhibitors (Thermo Fisher Scientific, A32957). Antibodies used for blotting were from Cell Signaling Technology including anti-phospho-473-Akt (catalog 4060); anti-Akt (catalog 2920); anti-phospho-p44/42 MAPK (ERK1/2) Thr202/204 (catalog 9101); and anti-p44/42 MAPK (ERK1/2) (catalog 4695). Antibodies used for immunostaining included anti-Talin (MilliporeSigma, T3287), anti-Vinculin (Thermo Fisher Scientific, 700062), anti-HS-1 (Cell Signaling Technologies, 3890), and anti-ARPC1B (MilliporeSigma, HPA004832). Alexa-488 or -647 Phalloidin were from Thermo Fisher Scientific. siRNA for ARPC1B was from Horizon Inspired Cell Solutions (catalog L-012082-00-0005). Lipopolysaccharide was from MilliporeSigma (catalog L6511). FITC-gelatin was from Thermo Fisher Scientific (catalog G13187). Fluo-8 AM was from Abcam (catalog ab142773). SYK inhibitors BAY 61-3606 (Selleck) or R406 (AdooQ Bioscience) were used at 500 nM.

### Statistics

Statistical analyses were performed using GraphPad Prism 6 with significance set at a minimum of *P* < 0.05. Analyses between 2 groups only were assessed using Student’s 2-tailed *t* test for normally distributed data or Fisher’s exact 2-tailed test for contingent values. Grouped data were analyzed using a 1-way ANOVA followed by a Dunnett’s post hoc test when compared with a single group, or Tukey’s multiple-comparison test when compared with all. Grouped data with 2 factors were analyzed using a 2-way ANOVA followed by Tukey’s or Holm-Sidak’s multiple-comparison test. The actin polymerization curves were analyzed as a plateau followed by 1-phase association.

### Study approval

Ethics for the use of human samples were approved by the Hospital for Sick Children (REB 1000024905).

## Author contributions

GL, YZ, AMM, and SAF designed the study. GL, YZ, PO, SM, PB, and SAF conducted experiments. GL, YZ, CG, AMM, DJM, and SAF analyzed and interpreted data. GL, YZ, AMM, and SF drafted the manuscript. All authors reviewed and approved the manuscript.

## Supplementary Material

Supplemental data

Supplemental video 1

Supplemental video 2

Supplemental video 3

## Version 1 10/21/2021

In-Press Preview

## Version 2 12/08/2021

Electronic publication
