# Supplementary material for: ARPC1B binds WASP to control actin polymerization and curtail tonic signaling in B cells
Source: JCI Insight. 2021 Dec 8;6(23):e149376. doi: 10.1172/jci.insight.149376 (PMC8675194; doi:10.1172/jci.insight.149376)
Supplement: Supplemental data [file jciinsight-6-149376-s047.pdf]

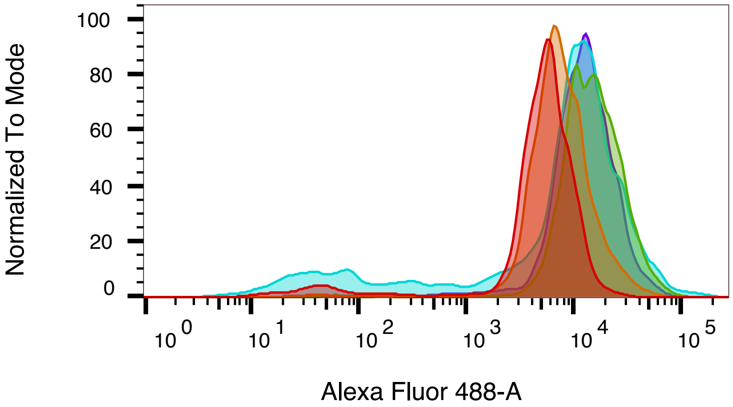

	Sample Name	Subset Name	Count
	B cells_P1 phall_004.fcs	Fixable Blue-A, Alexa Fluor 488-A subset	1722
	B cells_P2 phall_006.fcs	Fixable Blue-A, Alexa Fluor 488-A subset	2559
	B cells_Dad phall_010.fcs	Fixable Blue-A, Alexa Fluor 488-A subset	2665
	B cells_Mom phall_012.fcs	Fixable Blue-A, Alexa Fluor 488-A subset	1675
	B cells_Ctl phall_014.fcs	Fixable Blue-A, Alexa Fluor 488-A subset	3275

Supplemental Figure 1. *F-actin polymerization by flow cytometry*. LCLs stained with F-actin in suspension. A representative histogram related to Figure 1C.

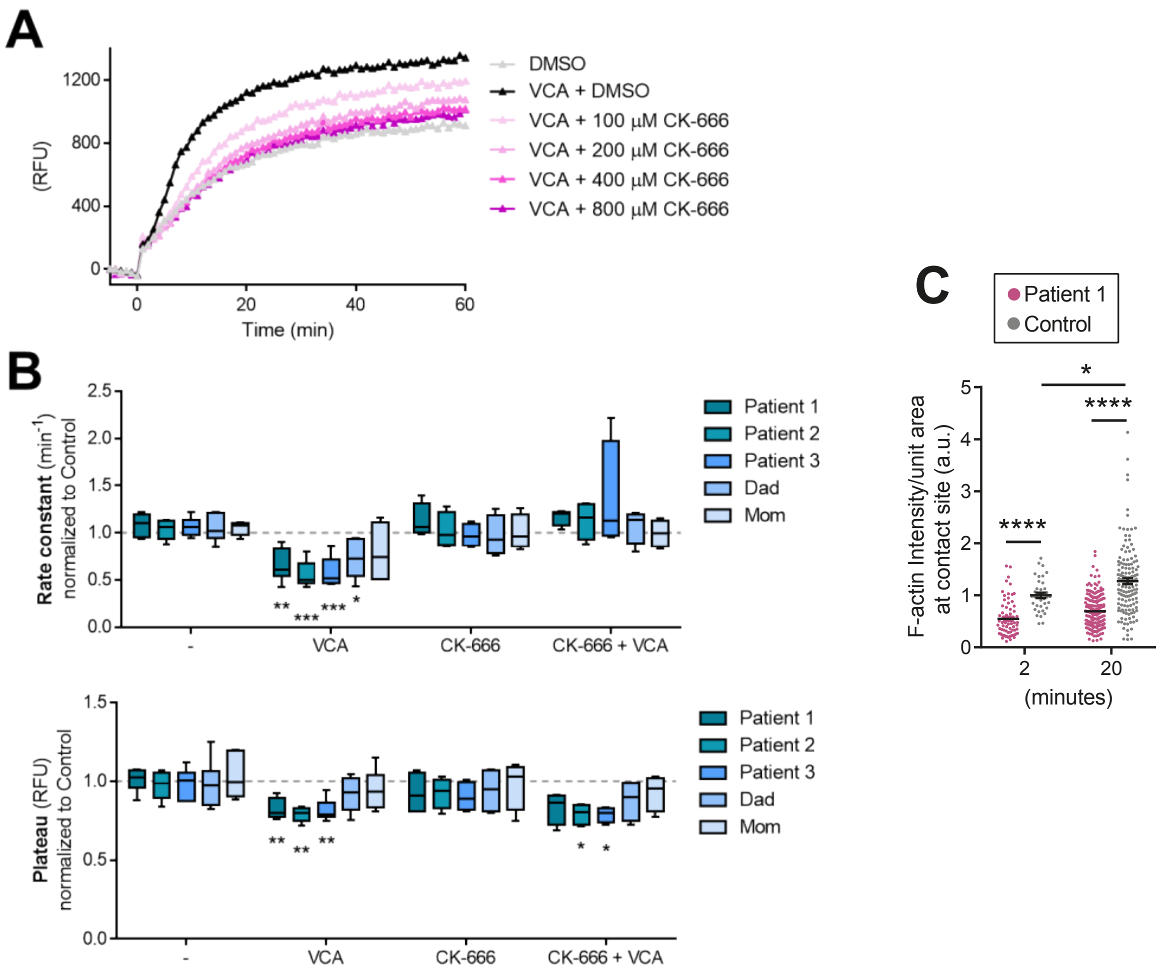

Supplemental Figure 2. *The Arp2/3 complex inhibitor CK-666 inhibits VCA-induced F-actin polymerization*. A) Actin polymerization assay in Control LCLs stimulated at t=0 min with the same concentration of recombinant VCA and increasing doses of CK-666 (n=1). B) Alternative analysis of data for Figure 2, where the rate constant and plateau for each treatment are normalized to the Control value. Data are represented as 2.5-97.5 percentile box plots (n=4-6) and analyzed using a 2-way ANOVA and Tukey's multiple comparison's test; significance represents a comparison to the Control sample within the same treatment group. C) LCLs seeded onto anti-IgG coated coverslips were

imaged at the contact surface by confocal microscopy. Each dot represents one field of >10 cells. Here and elsewhere in the supplemental files, *p<0.05, **p<0.01; ***p<0.001.

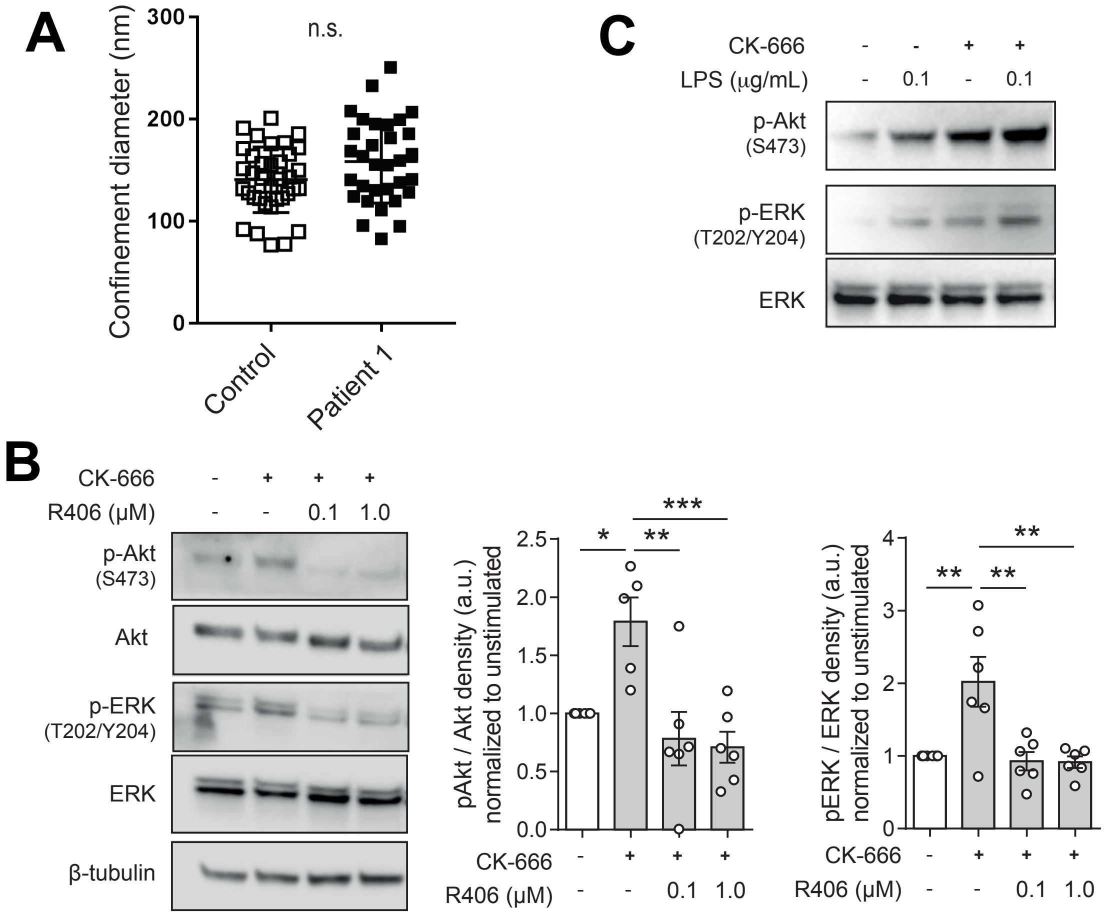

Supplemental Figure 3. *Inhibition of ARP2/3 does not change confinement size despite decreasing the number of confined BCRs and sensitizes TLR4 signaling*. A) The median confinement diameter for tracks defined as confined in Figure 5H. B) Ramos cells at indicated times after addition of CK-666. Results were assessed using a 1-way ANOVA and Dunnett’s test comparing all groups to the DMSO control, n=6. B) Ramos

cells treated with CK-666 for 15 min +/- the SYK inhibitor R406 and blotted for AKT and ERK phosphorylation. Results were assessed using a 1-way ANOVA and Dunnett's test (n=6). All column data are represented as the mean ± SEM. C) Primary B cells given CK-689 (control) or CK-666 for 2 h before stimulation with 100 ng/mL LPS. Representative blot shown. n=3.

Supplemental Video 1. *Surface IgG-BCR lateral mobility in LCLs*. Representative real-time videos of IgG-BCR mobility in unstimulated Control (left) and Patient 1 (right) LCLs.

Supplemental Video 2. *Cytosolic calcium oscillations in resting B cells*. Fluo-8 signal over time of control Ramos cells (left) and cells treated with CK-666 for 20 min (right) subsequently imaged for 5 min.

Supplemental Video 3. *Cytosolic calcium oscillations in ARPC1B-KO B cells*. Fluo-8 signal over time of WT Ramos cells (left) and ARPC1B-KO cells (right) imaged for 5 min.
